# Management of Fall Armyworm (*Spodoptera frugiperda*) Through Combined Plant Extracts and Microbial Biocontrol Agents

**DOI:** 10.3390/insects17010110

**Published:** 2026-01-19

**Authors:** David P. Tokpah, Ovgu Isbilen

**Affiliations:** 1 Department of Bioengineering, Faculty of Engineering, Cyprus International University, Northern Cyprus, Mersin 10, 99258 Nicosia, Turkey; oisbilen@ciu.edu.tr; 2 Biotechnology Research Center, Cyprus International University, Northern Cyprus, Mersin 10, 99258 Nicosia, Turkey; 3 Department of Natural Resource Management, Central Agricultural Research Institute (CARI), Suakoko P.O. Box 3929, Liberia; 4 Department of Pharmacy, Faculty of Pharmacy, Cyprus International University, Northern Cyprus, Mersin 10, 99258 Nicosia, Turkey

**Keywords:** bacterial isolates, bio-extracts, biocontrol agents, fall armyworms

## Abstract

The fall armyworm (FAW), *Spodoptera frugiperda*, poses a major threat to global maize production. Reliance on synthetic pesticides has contributed to pest resistance and environmental degradation, underscoring the need for sustainable alternatives. In this study, ethanolic extracts of neem (*Azadirachta indica*) and moringa (*Moringa oleifera*), together with maize-associated bacterial isolates, were evaluated for their biocontrol potential against fall armyworm. Gas chromatography-mass spectrometry (GC-MS) analysis for bioextract identification revealed tissue-specific chemical diversity, identifying eight key phytochemicals, including *octadecanoic acid*, *trimethyl fluorosilane*, and *hexadecanoic acid* in neem, and *trimethyl fluorosilane*, *ethyl oleate*, *ethyl (9Z*,*12Z)*, *octadecanoic acid*, and *benzenedicarboxylic acid* in moringa extracts. Eighty-nine bacterial isolates were screened for extracellular enzyme activities (cellulase, chitinase, glucanase, and protease) and siderophore production, among which four strains, DR-55 (*Bacillus subtilis*), HL-7 (*Bacillus cereus*), HL-37 (*Bacillus cereus*), and DR-63 (*Enterobacter* sp.), exhibited >50% biocontrol efficacy under greenhouse conditions. A strong correlation (r = 0.88) was observed between in vitro antagonistic activity and greenhouse performance, validating the screening approach. Fall armyworm mortality was the highest in larvae (up to 80%), moderate in pupae (15–17%), and the lowest in adults (6–7%), respectively. Overall, plant bio-extracts and maize-associated microbial isolates represent a promising, non-hazardous strategy for sustainable fall armyworm management while preserving maize plant health.

## Simple Summary

The fall armyworm (*Spodoptera frugiperda*) poses a significant threat to maize production, particularly in Africa, where smallholder farmers often lack access to safe and effective control measures. The overuse of chemical insecticides has led to an increase in the generation of resistance and caused environmental health problems. A study evaluating the integrative effect of neem (*Azadirachta indica*) and moringa (*Moringa oleifera*) bio-extracts and associated maize bacteria as a novel sustainable solution for fall armyworm control was performed. Chemical profile stapling of the two plant extracts and screening for enzymatic properties known to be associated with biocontrol activity in bacterial isolates was also performed. The treatments were most effective against the larval stages, followed closely by pupae, and least effective on adults. These results suggest that plant-based extracts and beneficial bacteria can support environmentally friendly FAW control when used as an option in an integrated pest management strategy focused on early larval stages. Future work will focus on evaluating the combined or possible synergistic effects of neem (*Azadirachta indica*) and moringa (*Moringa oleifera*) bio-extracts and bacterial isolates with high biocontrol efficiency on FAW under greenhouse and field conditions.

## 1. Introduction

Fall armyworm (FAW), *Spodoptera frugiperda* (*Lepidoptera*: *Noctuidae*), is one of the most polyphagous insect pest species on record, affecting over 350 plant species in more than 70 botanical families [1,2]. One of its favorite hosts is maize (*Zea mays*), making FAW a significant threat to food security and rural livelihoods in sub-Saharan countries, where maize is a major staple crop. Having invaded Africa in 2016, FAW has spread rapidly throughout the continent and is indeed prevalent in West African countries such as Liberia, resulting in significant losses that range between 20% and >50% under severe infestation [3,4,5,6]. The pest’s high fecundity, migratory habits, and potential to infest a range of agroecological zones have added to the complexity of its control, making FAW an almost regional (if not global) threat to agriculture [6]. Effective and sustainable pest control measures are therefore necessary to minimize their economic, ecological, and social repercussions.

Currently, FAW management techniques predominantly depend on synthetic insecticides. Although these products can deliver immediate control, their widespread and somewhat indiscriminate application has been associated with several notable limitations, including insect resistance to insecticides, soil and water resource contamination, and off-target impacts on non-target organisms (e.g., pollinators and natural enemies), among others [7,8]. FAW populations are already resistant to the most used chemical classes (e.g., pyrethroids and organophosphates) in some regions, and concerns over the long-term sustainability of such control measures have been raised [9,10]. The inadequate availability and substandard use of personal protective equipment in smallholder farming systems contribute to additional pressure on human health. Such concerns have amplified the worldwide demand for ecologically sound options that are well-suited for integrated pest management (IPM) systems.

Biopesticides, from microbial sources and plants, are one such alternative attracting attention amid renewed interest, as these appear to be more environmentally friendly, locally available, and with low toxic effects to humans. Neem (*Azadirachta indica*) and moringa *(Moringa oleifera*) are the two most extensively studied botanical resources for pest management in tropical and subtropical regions [11]. Neem contains a variety of aglycones known as limonoids, with azadirachtin playing a role in such hormone-based interference with feeding, molting, and replication, which prevents damage to crops without causing immediate toxicity to insects [12]. Aside from nutritional and medicinal value, moringa also exhibits secondary metabolites that have insecticidal, antifeedant, and repellent properties [13,14]. Several studies have reported the efficacy of pesticides that are neem and moringa-based products on lepidopterans such as FAW, but their effectiveness is apparently stage-dependent, and the majority achieve suboptimal success when used alone under field conditions [15,16].

Concurrently, plant-associated bacteria living on plants in a natural context are becoming attractive candidates for biocontrol. Endophytic and rhizosphere bacteria are also able to inhabit plant tissues and contribute to plant defense in several ways. The production of chitinases, proteases, and glucanases, as well as the production of siderophores that deprive pests of nutrients and bio-extract secondary metabolites [17,18], are also attractive candidates for biocontrol. Several bacterial genera, such as *Bacillus*, *Pseudomonas*, and *Enterobacter*, have been recognized to inhibit insect pests directly with an *entomopathogenic* activity or indirectly with a plant resistance stimulation [19,20]. Of particular interest may be those that occur in natural association with maize and thus under selection for host physiology, agricultural practices, and other ecophysiological factors, which are compatible with the native host microbiota.

Despite increasing evidence of the involvement of plant-associated bacteria in pest regulation, many questions are still unanswered. First, most of the research has only examined a few taxa or single plant compartments, usually emphasizing rhizosphere isolates and ignoring phyllosphere and internal tissue-associated communities. Second, the reasoning behind in vitro bacterial screening (for example, enzyme production) and its relation to in vivo pest suppression may not be so evident. Third, neither neem nor moringa combined bio-extracts nor microbial biocontrol agents have been tested against FAW; however, research focused on evaluating the integrative effect of neem (*Azadirachta indica*) and moringa (*Moringa oleifera*) bio-extracts and associated maize bacteria as a novel sustainable solution for fall armyworm control. Such evaluation may be particularly beneficial, as plant natural products can reduce defenses of insects [21,22] and make them more vulnerable to microbial antagonists, but vice versa, bacteria can improve the longevity and efficacy of botanical treatments.

Thus, this study was conducted to (i) identify bioactive compounds in neem and moringa extracts, employing gas chromatography mass spectrometry (GC-MS); (ii) screen maize-associated bacterial isolates from leaves, bark, and root parts of the plant to inventory them for enzymatic and antagonistic features associated with a potential for biocontrol; (iii) evaluate combined effects of chosen plant extracts and bacterial isolates on FAW under greenhouse and field conditions; and (iv) assess relationships between laboratory-based screening variables correlated with in vivo control of the pest. Through the fusion of both phytochemical and microbial approaches, this study aims to develop a sustainable, chemical-free strategy for managing FAW and to highlight maize-associated bacteria as an underexplored resource that can be leveraged for pest control.

## 2. Materials and Methods

### 2.1. Site and Experiment Design

The experiments were carried out at the Central Agricultural Research Institute (CARI), Bong County, Liberia (7°00′ N 9°34′ W; altitude 245 m) with a tropical climate (mean annual rainfall of 2195 mm and temperature of 27 °C), where precipitation influences agricultural development and pest proliferation [23]. The soil in the experimental field was assessed and determined to be neutral (pH 7.0) with low organic matter (2.7%), nitrogen (0.24%), phosphorus (67.4 mg/kg), and potassium (0.44 cmol/kg), alongside moderate mineral content. The soil composition consisted of 40% sand, 42% silt, and 18% clay, categorizing it as loam, a type prevalent in CARI [24]. Maize (*Zea mays*) plants were used in a randomized complete block design with blocks spaced 120 cm apart, plots spaced 90 cm apart, and plants at 60 cm distance to give nine plants per replicate.

### 2.2. Preparation of Plant Bio-Extracts

Fresh leaves of *Azadirachta indica* (neem) and *Moringa oleifera* were collected from the CARI experimental field. The plant materials were washed, air-dried at room temperature for 48–72 h, and processed into fine powder. An in-laboratory extraction assessment technique was developed to evaluate the bio-extract of each plant component before formulation.

A total of six treatments (T1–T6) were prepared for the experiment. To formulate the treatments, 2–10 mL of ethanol and 20–100 mg of dried neem and moringa material were mixed by adding 90–98 mL of distilled water (D.H_2_O) and subsequently brought to a final volume of 100 mL. This systematic preparation of solutions with varying compositions allowed for an exploration of a spectrum of ethanol concentrations, bio-extract quantities, and dilution levels. All prepared solutions were stored at 4 °C before application.

Treatment Preparation

T1: 10 mL bio-extract + 20 mg plant material + 90 mL D.H_2_O;
T2: 8 mL bio-extract + 40 mg plant material + 92 mL D.H_2_O;
T3: 6 mL bio-extract + 60 mg plant material + 94 mL D.H_2_O;
T4: 4 mL bio-extract + 80 mg plant material + 96 mL D.H_2_O;
T5: 2 mL bio-extract + 100 mg plant material + 98 mL D.H_2_O;
T6 (Control): Distilled water only (100 mL).

The formula is as follows:

$$\mathrm{Concentration}\;(\mathrm{mg}/\mathrm{mL})\;=\frac{\mathrm{amount}\;\mathrm{of}\;\mathrm{bio}-\mathrm{extract}\;(\mathrm{mg})}{\mathrm{total}\;\mathrm{volume}\;(\mathrm{mL})}$$

### 2.3. Gas Chromatography-Mass Spectrometry (GC-MS) Analysis for Bio-Extract Identification

An assessment was conducted to review potential bio-extracts, evaluating each component for its bioactive compounds. Gas Chromatography-Mass Spectrometry (GC-MS) analysis of the whole plant extract of *Moringa oleifera* and neem was performed using the equipment Thermo GC-Trace Ultra Version: 5.0 (Thermo Fisher Scientific, Milan, Italy), Thermo MS DSQ II. The equipment features a DB-35-MS (Agilent Technologies, Santa Clara, California, USA) Capillary Standard non-polar column with dimensions of 30 mm × 0.25 mm ID × 0.25 μm film. The carrier gas used was helium with a flow of 1.0 mL/min. The injector was operated at 250 °C, and the oven temperature was programmed as follows: 60 °C for 15 min, then gradually increased to 280 °C for 3 min. The identification of components of each plant extract was based on the Wiley and NIST libraries, as well as the comparison of their retention indices. The constituents were identified after comparison with global standards previously tabulated by [25,26], those available in the computer library (NIST and Wiley) attached to the GC-MS instrument, and the results obtained have been tabulated. We conducted three separate trials to confirm that the results were repeatable.

### 2.4. Bacterial Isolation and Preparation

Sample preparation for the isolation of plant-associated microorganisms was as per [27,28]. We washed the roots in water, removing the soil while underwater, until they were clear-washed three times with sterile water. We also sterilized the straws and leaves in water. Three grams of each root, leaf, and stem sample were placed in sterile Erlenmeyer flasks (50 mL, borosilicate glass; Pyrex^®^, Corning Inc., Corning, NY, USA; Cat. No. 4980-50) with 27 mL of sterile PBS solution (1.2 Na_2_HPO_4_; 0.18 NaH_2_PO_4_; 8.5 NaCl), g/L^−1^, at pH 7.6, and homogenized using sterilized mortar and pestles separately. Additionally, we included 27 mL sterile water with sterile glass beads per sample to ensure that the samples were not pulverized.

To isolate the bacterial population, homogenized samples were shaken (180 rpm) for 30 min and left to stand for 5 min. After sedimentation, 0.1 mL of the supernatant was serially diluted with aliquots to (10^−4^–10^−6^) ml, smeared on an R_2_A medium plate, and inoculated at 28 °C for 48–72 h to obtain cultures (form, color, and texture) having bacteria from CFU 50–300. All the colonies were then counted after incubation, all of them being picked up, purified, and stored as previously described by [29,30].

For in vitro isolation, each (leaf, stem, and root) sample was submerged in 1% sodium hypochlorite for five minutes, then soaked for two minutes in 70% alcohol before rinsing three times with sterile water. We ground 3.0 g of each sample in sterilized mortars and pestles with the addition of 27 mL of sterile water. Subsequently, 10^−1^, 10^−2^, and 10^−3^ dilutions were prepared to smear 0.1 mL of each extract aseptically on R_2_A plates. Batch cultures of bacteria, grown at 28 °C for 48 h, were repeated four times. Following the method [31,32,33], all the samples contained in the plates were counted and picked to achieve separation and purification, followed by storage on slants with 48% glycerol at −70 °C.

### 2.5. Extracellular Hydrolytic Enzyme Activities and Siderophore Screening

Cellulase activity was determined using CMC agar (1.0%, *w*/*v*) after incubation at 28 ± 2 °C for 48–72 h, and the plates were stained with 0.1% Congo red and destained with 1 M NaCl, from which hydrolysis zones were measured (mm). The activity of cellulases in CMCPRU was measured quantitatively by incubating the crude enzyme extracts with 1.0% (*w*/*v*) CMC in 50 mM sodium citrate buffer (pH 5.0); then, the reaction mixture was further subjected to the DNS assay, and readings were taken at 540 nm after an incubation period of 30 min at 50 °C. A total of 1U of activity was described as 1 µmol glucose min^−1^ mL^−1^ released. Commercial cellulase and native-substrate-only reactions were used as positive and negative controls, respectively [34].

Colloidal chitin (0.5–1.0%, *w*/*v*) was used for assaying chitinase enzyme activity. The isolates were grown on chitin agar at 28 ± 2 °C for a period of three to five days, and the diameters of halos were recorded. The enzyme extracts were incubated with colloidal chitin in 50 mM sodium acetate buffer (pH 5.5) at 37 °C for 30 min, and the liberated N-acetyl glucosamine was determined by the DNS method (540 nm). Activity was given as IU mL^−1^ with *S. marcescens* as the positive control [35].

Protease production was evaluated by measuring clear zones (mm) on skim milk broth plates (1.0–2.0%, *w*/*v*) after incubating cultures at 30–37 °C for 24–48 h. Quantitative studies were performed using 1.0% casein in 50 mM phosphate buffer at pH 7.5 for 30 min at 37 °C, followed by Folin–Ciocalteu analysis at A660 nm. One unit of protease activity was defined as the amount of enzyme required to release 1 µg of tyrosine min^−1^ mL^−1^, with Bacillus subtilis serving as a positive control [36].

The β-1,3-glucanase activity was estimated by using laminarin (0.5–1.0%, *w*/*v*) prepared in sodium acetate buffer 50 mM (pH 5.0), which was incubated with enzyme extracts at 37–50 °C for 30–60 min. Reducing sugars were measured by DNS assay at 540 nm, and activity was expressed in IU mL^−1^. The positive control was provided by commercial β-1,3-glucanase [37].

Siderophore production was determined using the Chrome Azurol S (CAS) test. Halogens were cultured on CAS agar buffered with 30 mM PIPES (pH 6.8) for 48–72 h at 28 ± 2 °C, and the diameters of halos were determined. A quantitative assessment was made by the CAS shuttle assay and the absorbance at 630 nm. To express the results, siderophore units (%) were calculated according to $(A_{r}-A_{s})/A_{r}\times 100$. Positive control was *Pseudomonas aeruginosa,* and negative control was uninoculated media [38].

### 2.6. Biocontrol Strains Assessment

We developed an evaluation technique in our laboratory to score biocontrol strains based on varying scores of antagonistic and enzyme-producing activities [39,40]. We established an assessment system to rate the biocontrol potential of all bacteria isolates based on in vitro activities of their extracellular metabolites under two categories: Category (A) consisted of 3 items (cellulase, glucanase, and chitinase), which accounted for 250, 148, and 44 antagonistic isolates, and Category (B) comprised 2 items (siderophores and protease), which accounted for 124 and 91 antagonistic isolates. We adopted a zero-to-three-point scale to score the isolates for enzyme activity, with 0 meaning no hyaline zone present. For the isolates under category (A), one, two, and three indicated the occurrence of hyaline zones with widths of 0–5, 5–6, and >6 cm, respectively. One, two, and three points indicated the occurrence of hyaline zones with widths of 0–3, 3–6, and >6 cm per enzyme activity for the isolates under Category (B). For every antagonist, the sum of its scores under the respective categories is its assessed biocontrol score (up to 15 points). In addition, we tested each isolate in three replicates. The percentage of antagonist isolates from a single origin was calculated as follows:

$$\mathrm{Antagonist}\;(\%)=\frac{\mathrm{Number}\;\mathrm{of}\;\mathrm{antagonists}}{\mathrm{Total}\;\mathrm{isolates}}\times 100$$

### 2.7. ARDRA and BOX-PCR Fingerprint Analysis and Identification of Bacterial Isolates

The genotypes of the bacterial isolates obtained for the study were analyzed using ARDRA and BOX-PCR fingerprinting to understand their population structure. For ARDRA analysis, 89 bacterial DNA samples were prepared using the Mini BEST Bacterial Genomic DNA Extraction kit (TaKaRa Biotechnology Co., Ltd. Dalian, China). The partial nucleotide sequence of the amplified 16S rDNA was determined using the following primers: L1494-1514 (reverse) 5′-CTA CGG (AG) TA CCT TGT TAC GAC-3′ and U8-27 (forward) 5′-AGA GTT TGA TC (AC) TGG CTC AG-3′ in an automated DNA sequencer [41]. Amplification was performed with a Peltier Thermal Cycler PTC-200 (Bio-Rad, Watertown, MA, USA) using an initial denaturation step at 94 °C for 5 min, and subsequently 35 cycles of denaturation at 94 °C for 1 min, annealing at 56 °C for 2 min with an extension at 72 °C for 2 min, followed by a final extension at 72 °C for 10 min. The PCR products (10 µL) were digested for 2.5 h using the restriction enzymes AluI and MspI. The restriction fragments were separated on a mix gel (1.5% agarose + 2.25% Synergel) running in 1.0 × TBE buffer at 80 V for approximately 5 h and then stained with ethidium bromide and photographed under UV transillumination. We repeated the experiment three times to verify the reproducibility of the results.

For BOX-PCR fingerprint analysis, we prepared 35 bacterial DNA samples using the Mini BEST Bacterial Genomic DNA Extraction kit. BOX-PCR was carried out as described by using the BOX A1R primer 5′-CTA CGG CAAGGC GAC GCT GAC G-3′. Amplification was performed with a Peltier Thermal Cycler PTC-200 (Biozym Diagnostic, Hessisch Oldendorf, Germany) using an initial denaturation step at 95 °C for 6 min and subsequently 35 cycles of denaturation at 94 °C for 1 min, annealing at 53 °C for 1 min, with an extension at 65 °C for 8 min, followed by a final extension at 65 °C for 16 min. A 5 µL aliquot of amplified PCR products was separated by gel electrophoresis on mixed gel (0.5% agarose + 0.75% Synergel) in 1.0 × TBE buffer at 120 V for 6 h, stained with ethidium bromide, and photographed under UV transillumination (Bio-Rad). We verified the reproducibility of the results in three independent experiments.

### 2.8. Greenhouse Experiment

Several strains were grown in LB medium for 24 h at 28 °C and 280 rpm; the cells were harvested by centrifugation, washed, and resuspended in sterile 0.85% NaCl. The cell suspensions were adjusted to 5 × 10^5^ CFU mL^−1^, and the inoculation was performed at the vegetative stage of maize using a hand sprayer. FAW mortality bioassays were performed with second-third instar *Spodoptera frugiperda* larvae (20 for each replicate) held in ventilated plastic cups and reared on fresh (untreated) corn leaves. Bacterial strains were sprayed directly for full-body coverage. Bioassays were conducted under a controlled environment (28 ± 2 °C, 70 ± 5% RH, photoperiod of 12:12 h light: dark). Leaf damage and mortality were rated on a 0–5 scale (0 = no damage; 5 = 75–100% leaf damage) at 24, 48 and 72 h post-treatment using standard methods [42,43,44].

Biocontrol efficacy and relative FAW mortality rate were calculated as follows:

$$\mathrm{Relative}\;\mathrm{FAW}\;\mathrm{mortality}\;(\%)=\frac{\sum \left(\mathrm{Affected}\;\mathrm{plants}\;\times \;\mathrm{treatments}\right)}{\mathrm{Total}\;\mathrm{plants}\;\times \;\max\;\mathrm{score}}\times 100$$

$$\mathrm{Biocontrol}\;\mathrm{efficacy}\;(\%)=\frac{{\mathrm{Mortality}}_{\mathrm{control}}-{\mathrm{Mortality}}_{\mathrm{treatment}}}{{\mathrm{Mortality}}_{\mathrm{control}}}\times 100\;$$

We applied treatments meticulously and assessed the presence of FAW in the greenhouse.

### 2.9. Field Experiment

Field experiments were carried out at the Central Agricultural Research Institute (CARI), Bong County, Liberia (7°00′ N, 9°34′ W; 245 m above sea level), in the vegetative stage of maize. There were three replicates of various treatments assigned to a completely randomized block structure, and each replicate constituted nine rows of ‘Abontem’ maize established in 120 cm plots. Five bioactive phytochemical extracts and a water-treated control were assessed; extract suspensions were prepared according to conventional bioextraction protocols. Larvae movement on neighboring plants was recorded, and host plant exposure to fall armyworms was evaluated weekly at each growth stage. The density of FAW was quantified by counting the number of larvae per plant per plot according to [45,46].

### 2.10. Data Collection and Statistical Analysis

**Figure 1:**
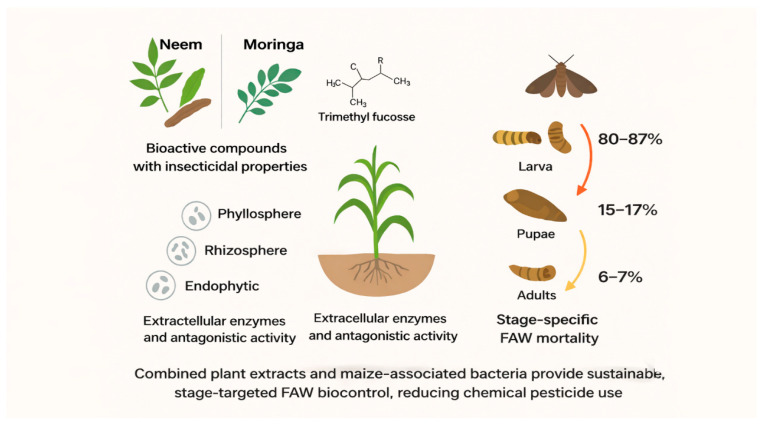
Graphical abstract concept.

Statistical analysis was performed in SPSS (version 7.05; IBM Corp., Armonk, NY, USA) or R software (R Foundation for Statistical Computing, Vienna, Austria). Correction of mortality figures was carried out by Abbott’s formula. Differences in treatment effects were tested by ANOVA, followed by Tukey’s HSD test to separate means at *p* < 0.05. Relationships of dose–response were evaluated with regression and correlations. Values of enzyme activities are presented as mean ± SE. Mortality, repellency, and the enzyme inhibition characters were presented in bar and line graphs (Figure 1).

**Figure 2:**
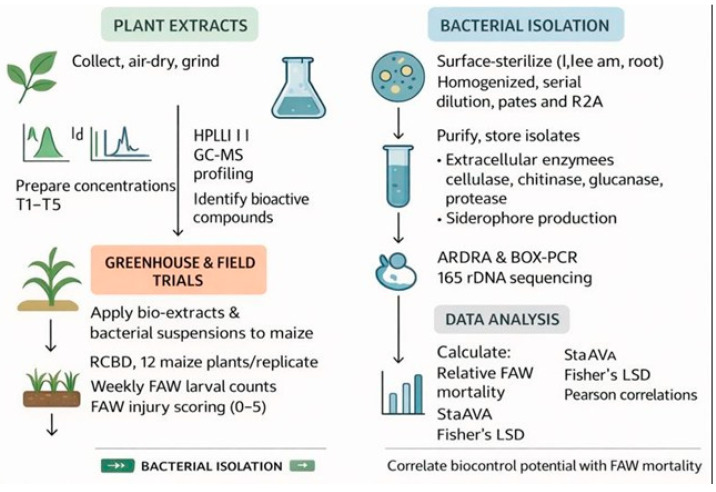
Integrated workflow for neem/moringa and biocontrol agent-based FAW management.

FAW population and biocontrol efficacy were assessed using standard scoring sheets. The densitometric analysis of ARDRA and BOX-PCR fingerprints, as well as the Pearson correlation, was determined. The clustering of isolates used UPGMA. Representative 16S rRNA sequences were subjected to BLAST (version 2.13.0+; National Center for Biotechnology Information [NCBI], Bethesda, MD, USA) in the NCBI database [47,48]. Contrast Mortality and Efficacy contrasts were tested by ANOVA (DPS v7.03) for mean separation by Fisher’s LSD test (*p* < 0). Similarly, the potential biocontrol efficacy relationship was estimated in JMP Pro 14 (Figure 2).

## 3. Results

### 3.1. GC-MS Analysis of Bio-Extracts

In neem (*Azadirachta indica*) and moringa (*Moringa oleifera*), three plant parts (root, bark, and leaf) were investigated for bioactive compounds. GC-MS profiling revealed several phytochemicals, of which eight principal bioactive compounds were identified in the ethanolic extracts of both species (Table 1). Five compounds were isolated from *M. oleifera*, while three were obtained from *A. indica*, and their identity was confirmed by comparison of their RT, MW, % peak area, and reported biological activity.

**Figure 3:**
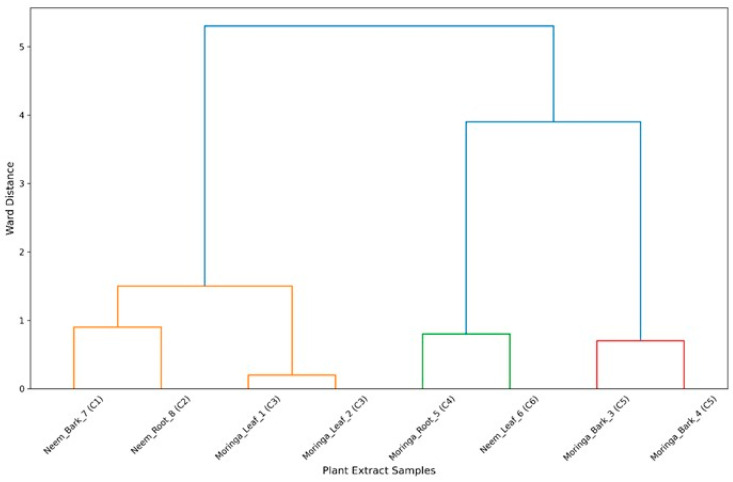
Hierarchical clustering dendrogram of GC-MS isolates from ethanolic extracts of *Moringa oleifera* and *Azadirachta indica*. Note: The dendrogram was generated using Ward’s linkage and Euclidean distance based on retention time, intensity, and peak area percentage. Each isolate is labeled with its source, plant part, and cluster number (C1–C6) at the base. Distinct clustering patterns reveal biochemical divergence between the leaf, bark, and root metabolites of both plant species.

The C1–C6 clusters in which the eight compounds were detected and categorized by retention time, peak intensity, and percentage area are presented in (Figure 3). Compounds of the same cluster presented closer chromatographic and quantitative characteristics. It was observed that *trimethyl fluorosilane* obtained from neem bark (C1) and *hexadecanoic acid* obtained from neem root (C2) were placed in separate clusters, which reflects their intensity and relative peak area as compared to others. A similar pattern was observed from the moringa leaves for *ethyl oleate* and *trimethyl fluorosilane*, grouped in cluster C3 due to similarities in spectral behavior and bioactivity profile. On the other hand, participants of moringa bark (*ethyl(9Z*,*12Z*) and *octadecanoic acid*) were together in C5, showing a similar constitution or operation. The corresponding dendrogram distinctly separates the interspecific and intratissue variation for the different metabolites, showing the biochemical diversity of both types.

### 3.2. Assessment of Extracellular Hydrolytic Enzyme and Siderophore Productions

The highest enzymatic potential was seen for phyllosphere and soil isolates (Table 2). Endorhizal and stem interior isolates were moderate enzyme producers, which might reflect a more specialized role for these bacteria in colonizing the internal tissues of plants. The assessment approved four potential isolates (DR-55, DR-63, HL-7, and HL-37) with a population of bacteria between 5.3 × 10^5^ to 6.5 × 10^6^ CFU/g on maize tissue, which were further evaluated in vivo (Table 2 and Table 3).

### 3.3. ARDRA and BOX PCR Analysis of Strains with Potential Biocontrol Efficacy

**Figure 4:**
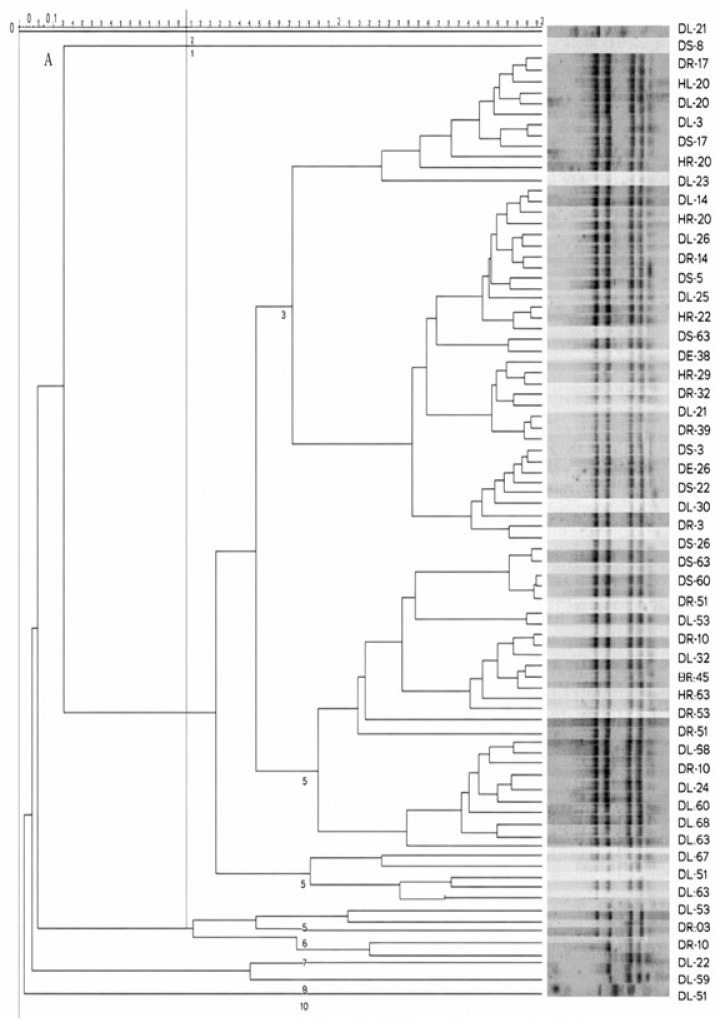
Amplified rRNA restriction analysis and ARDRA fingerprints. Note: ARDRA genomic fingerprints and enhanced rRNA restriction analysis are displayed. We created the dendrogram using GelCompar^®^ II version 4.5 (Applied Maths BVBA). We applied the Pearson correlation to the densitometric curves referred to by [49]. We then carried out the analysis using the unweighted pair-group method using arithmetic averages (UPGMA) for clustering analysis.

**Figure 5:**
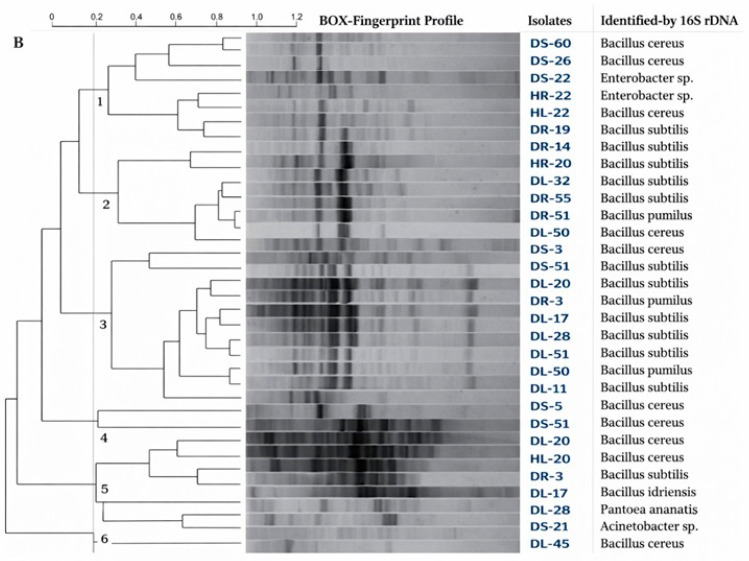
Amplified rRNA restriction analysis and BOX-PCR genomic fingerprints. Note: BOX genomic fingerprints and enhanced rRNA restriction analysis are displayed. We created the dendrogram using GelCompar^®^ II version 4.5 (Applied Maths BVBA). We applied the Pearson correlation to the densitometric curves referred to by [50]. We then carried out the analysis using the unweighted pair-group method using arithmetic averages (UPGMA) for clustering analysis.

Out of 720 bacterial isolates, 89 were chosen because they had a score greater than 2. They were then put through ARDRA/BOX fingerprint analysis to eliminate repetition in the subsequent study. The isolates were categorized into 10 clusters based on 65% similarity (Figure 4). Cluster 1 included 1 isolate, clusters 2, 7, 9, and 10 contained 2 isolates each, and clusters 6, 8, and 9 contained 4, 29, and 47 isolates, respectively (Figure 4). Thirty-five bacterial strains with the highest evaluation scores were selected from each cluster. A BOX-PCR experiment was conducted to reorganize them into six clusters exhibiting significant diversity (Figure 5). We amplified portions of the 16S rRNA gene from the genomes of these 35 bacterial species.

### 3.4. Effectiveness of Biocontrol Agents Against Fall Armyworm (FAW) Under Greenhouse

Out of 35 bacterial isolates tested, four strains (DR-55, DR-63, HL-7, and HL-37) exhibited more than 50% biocontrol activity against FAW (Table 4). Based on 16S rDNA sequences, strain DR-55 was *Bacillus subtilis*, whereas HL-7 and HL-37 were identified as *B. cereus*, and strain DR-63 belonged to *Enterobacter* sp. These identifications were supported by ARDRA and BOX PCR analysis.

### 3.5. Correlation Between Assessment Scores and Biocontrol Efficacy

**Figure 6:**
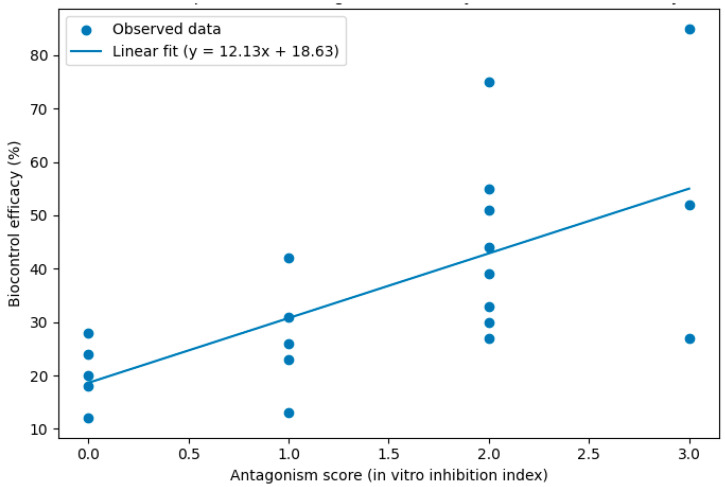
Correlation between antagonism score and biocontrol efficacy (%). Note: Each point represents an individual isolation, with the red line indicating the linear regression. A moderate positive correlation was observed (r=0.57), suggesting that higher antagonistic activity is generally associated with improved biocontrol efficacy against *Spodoptera frugiperda*.

**Figure 7:**
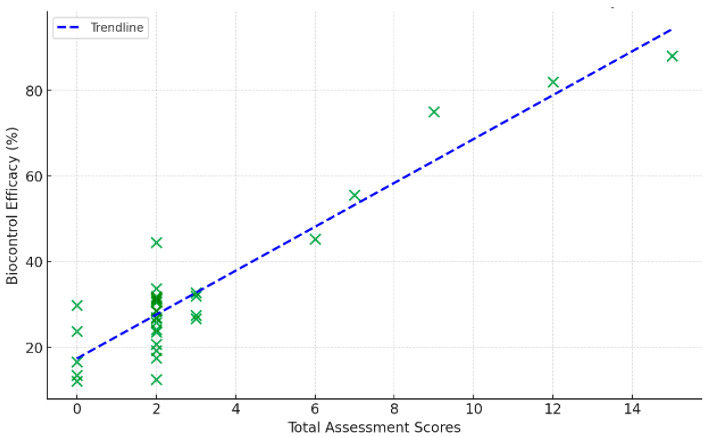
Correlation between assessment scores and biocontrol efficacy (%). Note: The scatter diagram indicates good correlation between scores and biocontrol efficacy. The Pearson correla-tion coefficient is r ≈ 0.88, suggesting a strong and statistically significant relationship. The slanted blue line is a linear fit and indicates that biocontrol effectiveness increases with the assessment score.

Pearson correlation analysis showed highly significant correlations between in vitro antagonistic tests and biocontrol efficacy (Table 5). In particular, the correlation coefficients of antagonistic tests and assessment scores (and greenhouse tests) were 0.57 and 0.88 (Figure 6 and Figure 7), indicating that higher assessment scores could predict biocontrol efficacy.

### 3.6. Effect of Plant Bio-Extract (Neem and Moringa) on Fall Armyworm

The stage-dependent efficacy of the treatments was observed for *S. frugiperda* (Table 6). Mortality was the highest in larval, moderate in pupal, and the lowest in adult stages, revealing that the larvae are more sensitive to the tested extract or compounds. Mortality induced by T3 concentration was found to be significantly higher in larvae (80%). Moderate larvicidal activity (13–31%) of T1 and T2, with low effect provided to pupae and adults T4 and T5 (8–10% and 2–3%, respectively). Control (T6): mortality was very close to zero through all stages, validating a low-level natural mortality and species-specific effect of the exposure (Table 6).

### 3.7. LC_50_ Determination and Stage-Specific Toxicity

**Figure 8:**
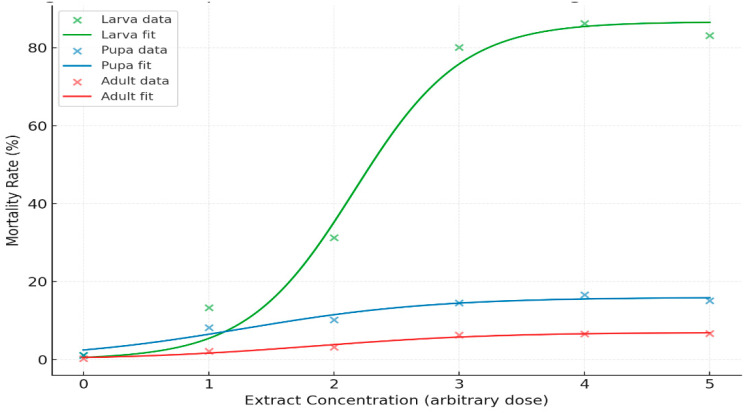
Mortality response of fall armyworm (FAW) to neem and moringa extract concentrations. Note: The fitted logistic dose–response curves for neem and Moringa extracts on FAW mortality rates are displayed. Each line represents the modeled response for larval, pupal, and adult stages, showing how mortality increases with extract concentration.

The logistic dose–response curves of the neem and moringa extract concentrations on the mortality of *S. frugiperda* in the larval, pupal, and adult stages are illustrated in (Figure 8). Mortalities increased with increasing extract concentration in all life stages, but the responses were stage-specific.

**Figure 9:**
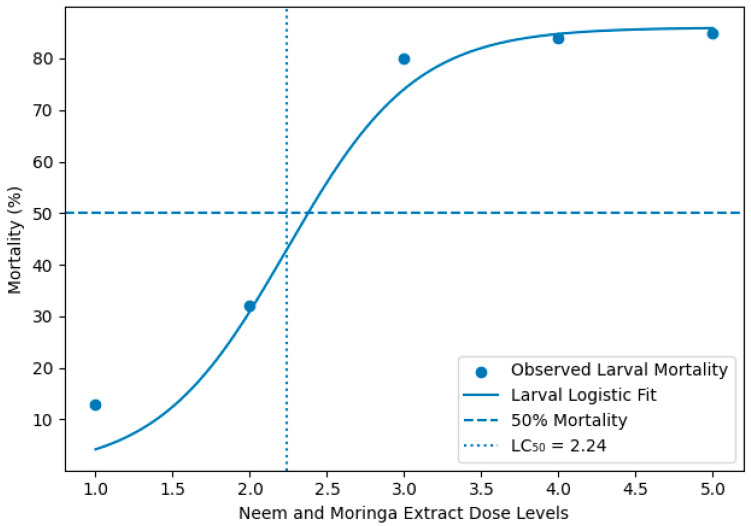
Logistic regression models and LC_50_ values for the larval stage. Note: Dose–response curve of *Spodoptera frugiperda* larvae exposed to combined neem (*Azadirachta indica*) and moringa (*Moringa oleifera*) is displayed. The open circles are the observed mortality, the solid line is the fitted logistic regression model. Thin dashed and dotted lines represent 50% mortality and the estimated LC_50_ (2.24), respectively.

**Figure 10:**
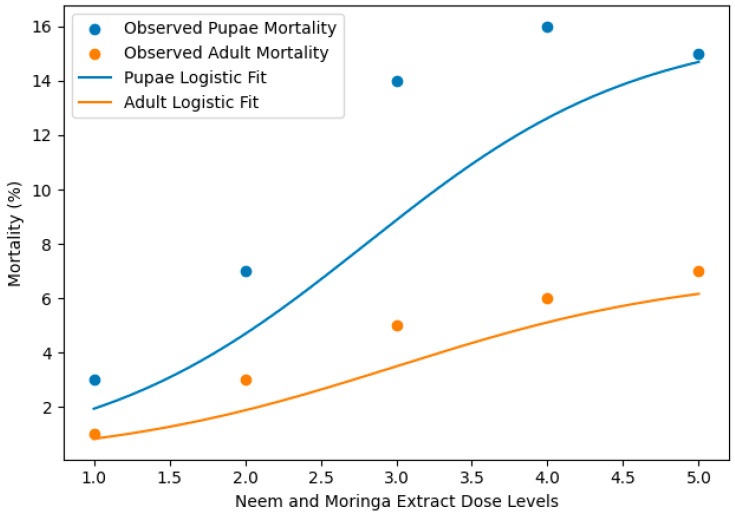
Logistic regression models and LC_50_ values for the pupae and adult stage. Note: Dose–response curves are displayed for the pupal and adult mortality of *Spodoptera frugiperda* after exposure to neem (*Azadirachta indica) and moringa* (*Moringa oleifera*) combined treatments. The points show the observed mortality, while the solid lines are fitted logistic regression models.

LC_50_ values at each stage of the *S. frugiperda* exposed to mixed neem (*Azadirachta indica*) and moringa (*Moringa oleifera*) extracts were calculated based on logistic dose–response models (Table 6). Larvae were the most sensitive stage, with LC_50_ 2.24 and >80% dead at higher concentrations (Figure 9). In contrast, pupal mortality was low (15–17%), and adult mortality was lower (6–7%) (Figure 10). In accordance with toxicological rules, we found a continuous decrease in susceptibility from pupae to adults.

**Figure 11:**
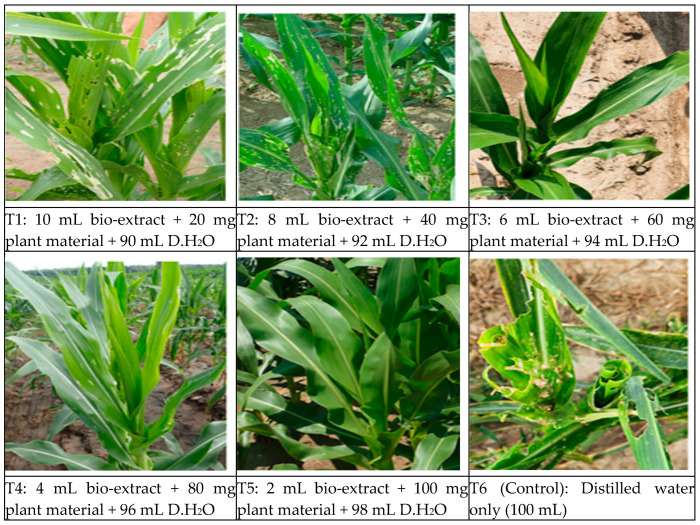
Maize leaves infested by FAW in the field. T1 and T2 observed moderate to severe FAW pressure, with visible defoliation that could compromise crop yield if left unmanaged. Early intervention at the larval stage would likely be most effective in reducing further damage. T3, T4, and T5 acknowledged the highest, most severe FAW pressure. The maize plants appear vigorous and healthy, with strong leaf development suggesting good growth conditions. T6 (Control): the damage appears extensively on multiple leaves, which reduces photosynthetic capacity and potentially affects plant growth.

Taken together, bio-extract compounds of neem and moringa combinations efficiently control FAW and improve maize physiology (Figure 11).

## 4. Discussion

This research shows the combined efficacy of neem (*Azadirachta indica*) and moringa (*Moringa oleifera*) bio-extracts and maize bacteria isolates against *Spodoptera frugiperda,* FAW. Results are consistent with and complement prior studies on plant bioactive compounds and microbial biocontrol agents.

FAW has seriously damaged crops not just in Liberia but also in other areas of the world [51]. Due to concerning reports of FAW caused by *Spodoptera frugiperda*, we decided to assess bio-extracts and potential antagonistic bacterial isolates to control FAW (*Spodoptera frugiperda*) on maize. The Central Agricultural Research Institute (CARI) experimental field, located in Bong County, Suakoko District, Liberia, served as the site of our experiment.

Eight major bioactive compounds found in neem (*Azadirachta indica*) and moringa root, bark, and leaf were observed from the GC-MS profiling of ethanolic extracts. This agrees with earlier studies, which also reported structurally diverse metabolites in both species, such as fatty acids, phenolics, terpenoids, and volatile organic compounds [52]. Five compounds were isolated from *M. oleifera*, while three were obtained from *A. indica*, and their identity was confirmed by comparison of their retention time, molecular weight, % peak area, and phytochemical activity.

The eight compounds were divided into clusters (C1–C6) by the method of hierarchical clustering using Ward’s method and Euclidean distance. The *trimethyl fluorosilane* of neem bark and the *hexadecanoic acid* of neem root were separated as clusters, probably because their spectral intensity values had high levels, and they showed different chemical profiles [53]. Comparable clusters have also been observed for other neem metabolites by [54], who noticed the tissue-specific clusters.

*Ethyl oleate* from moringa leaf and *trimethyl fluorosilane* formed a cluster at C3, pointing to similar retention behavior [55]. On the other hand, the moringa bark metabolites *ethyl (9Z*,*12Z)*-*octadecadienoate* and *octadecanoic acid* were grouped in C5, indicating their common biosynthetic pathway. The dendrogram showed evident intraspecific and interspecific phytochemical differences, in agreement with previous work that had high chemical diversity between tissues of medicinal plants [55,56].

Enzymatic activities of extracellular hydrolytic enzymes (cellulase, chitinase, glucanase, protease, and siderophores), which investigate the antagonistic efficacy of these bacterial isolates, were measured as shown. *Phyllosphere* and soil-derived isolates also displayed the greatest enzyme activities, perhaps due to these isolation sources being constantly exposed to highly competitive microflora. In these environments, high production of extracellular enzymes may provide competitive advantages due to the factors involved in nutrition and antagonism against other microorganisms, including insect-associated ones. Similar reports were documented for phyllosphere and soil bacteria, in which strong microbial competition imposes or evolves the broad enzymatic properties [57]. On the other hand, isolates that were endorhizal and found inside the stem of plants showed intermediate enzymatic activity, indicating a more specialized ecological role linked to penetration of organs or tissues within the plant (colonization), since extensive hydrolytic activity could be limited by host regulation mechanisms. Although extracellular enzymes are known to mediate a wide range of microbial interactions and influence plant health, studies to date that have demonstrated this relationship have focused on general principles [58,59], whereas here we directly compare isolates from different maize tissues, showing that functional divergence is dependent upon tissue.

The multistage screening system established in this study, using ARDRA, BOX-PCR, and 16S rRNA gene analysis methods combined with greenhouse validation, is consistent with those documented for biocontrol agents studied (and references therein), but this investigation also represents several exciting differences. Molecular fingerprinting methods have also been frequently applied to eliminate redundancy of large bacterial collections and preserve intraspecific variation before biocontrol (e.g., punctual Ardra-based grouping followed by clustering via rep-PCR). However, in many previous studies, one genotyping method was applied, leading to replicates of testing the same closely related strains and increasing the number of experiments. Instead, we used a combination of ARDRA and BOX-PCR as performed in the work by [60], which facilitated greater selective pressure for isolate selection while maintaining genetic variation, thus optimizing screening efficiency.

The predominance of *Bacillus* spp. [61] is among isolates recorded with higher efficacy and in line with various other findings in which *Bacillus* species have been recognized as effective biocontrol agents against lepidopteran pests, including FAW. These taxa are very known to produce insecticidal metabolites, lytic enzymes, and spores that provide persistence both under greenhouse and field conditions. Similarly, the *Enterobacter* sp. was also detected in this study with >50% biocontrol efficacy, supported by recently emerging evidence [62,63] that non-*Bacillus* rhizobacteria can also significantly contribute towards FAW suppression. Furthermore, the actions of four biocontrol strains against FAW showed a substantial difference, exhibiting a correlation of more than 50% with the strains’ assessment scores. Moreover, the correlation between antagonistic tests and assessment scores in greenhouse efficacy is acknowledged as r = 0.57–0.88%, referencing a stronger correlation than those of many other screening studies that have substantial non-in vitro vs. in vivo correlations observed for variation or inconsistency among field evaluations. This result is consistent with multi-criteria assessment systems, which have better correlation to field efficacy than single antagonism control [64,65].

Such a relationship between the performance of biocontrol and the environment seen here is consistent with previous studies that reported temperature, soil type, and colonization niche largely affect bacterial persistence and insecticidal activity [66,67]. The differential performance of genetically different but functionally equivalent isolates is consistent with studies for other FAW agents [66,67].

FAW mortality dynamics showed a distinct stage-dependent susceptibility, with the larvae having the highest mortality (>80%), followed by pupae (15–17), whereas adult mortalities were low (6–7). This resistance was also modeled using the logistic model, which showed an increase in LC_50_ values from pupae to adults as a reflection of lower susceptibility during the insect maturation. Pupal and adult mortalities increased only slightly in relation to extract concentration. The pupal mortality never exceeded 17%, and adult mortality was always less than 7% at all dose levels tested, which indicated much lower susceptibility in comparison with larval stages. Similar development stage-related reactions to neem- or moringa-based treatments against lepidopteran pests have been described and are often associated with the thinner cuticle of larvae, higher rates of feeding, and greater larval metabolic activity during early stages [68,69]. Significantly, the application of plant extracts and bacterial isolates together resulted in a greater larval suppression than what has been standardized so far with these single-agent treatments, implying additive or synergistic rather than additive or overlapping effects.

The infusion of neem and moringa extracts and some bacterial isolates is thus a safe, ecologically acceptable approach to FAW control. Feeding experiments showed that untreated larvae killed leaves and caused whorl collapse and leaf abscission, while combined-treated plants had little visible injury. Although most previous studies have focused on the performance of plant-based insecticides or microbial biocontrol agents separately [70,71], this study indicates that combined neem and moringa have good efficiency, especially against FAW larvae. Furthermore, the strong positive correlations that existed between enzymatic activity, in vitro antagonism, bio-extracts, and FAW mortality highlight the utility of integrated screening programs to select effective biocontrol agents.

This study indicated that neem (*Azadirachta indica*) and moringa (*Moringa oleifera*) bio-extracts and maize-associated bacterial isolates can be a powerful, cost-effective strategy to manage *Spodoptera frugiperda*, an economically sustainable technology. GC-MS analysis revealed standardized phytochemical confirmations and profiles identical to all the extracts tested, whereas enzymatic and molecular (ARDRA and BOX-PCR) characterization discovered highly effective isolates, even genetically diverse genera like *Bacillus* spp. FAW mortality was stage-specific, and larvae were the most sensitive, whereas growth in the presence of plant-microbe treatments significantly exceeded that obtained with single-agent applications. A strong correlation among enzyme activity, in vitro antagonism, and greenhouse and field bio-efficacy results supports the validity of the multi-criteria screening method adopted, substantiating potential inclusion of both bio-extracts and microbial agents in sustainable FAW management programs. Future work will focus on evaluating the combined or possible synergistic effects of neem (*Azadirachta indica*) and moringa (*Moringa oleifera*) bio-extracts and bacterial isolates with high biocontrol efficiency on FAW under greenhouse and field conditions.

## Figures and Tables

**Table 1:** GC-MS spectral analysis of the ethanolic extract of neem and moringa.

Source	Plant Part	RT (min)	Intensity	Compound Name	Molecular Formula	Molecular Weight (g/mol)	Peak Area (%)	Biological Activity
Moringa	Leaf	35.42	216,950	*Trimethyl fluorosilane*	C_3_H_9_FSi	92.19	1.67	Insecticidal
Moringa	Leaf	36.25	216,950	*Ethyl oleate*	C_20_H_38_O_2_	310.51	1.56	Insecticidal
Moringa	Bark	40.43	23,120	*Ethyl(9Z*,*12Z)*	C_20_H_36_O_2_	308.50	1.64	Insecticidal
Moringa	Bark	41.45	2365	*Octadecanoic acid*	C_18_H_36_O_2_	284.48	1.00	Antibacterial
Moringa	Root	40.63	220,990	*Benzenedicarboxylic acid*	C_8_H_6_O_4_	166.13	31.69	Pesticidal
Neem	Leaf	45.60	23,560	*Octadecanoic acid*	C_18_H_36_O_2_	284.48	2.30	Antibacterial
Neem	Bark	50.00	25,432	*Trimethyl fluorosilane*	C_3_H_9_FSi	92.19	3.45	Insecticidal
Neem	Root	55.34	26,745	*Hexadecanoic acid*	C_16_H_32_O_2_	256.43	3.66	Pesticidal

Note: Bio extracts evaluated by WILEY7. The LIB data library is present in the GC-MS. Cluster membership interactive table showing each isolate, compound, RT, intensity, peak area %, and assigned cluster.

**Table 2:** Antagonistic ability and metabolic enzyme activities of bacterial isolates screened from different parts of maize samples.

Strain Source	Time ^a^	Bacteria Concentration (CFU/g) ^b^	Number of Bacteria Strains	Number of Isolates with Antagonism Ability and Metabolite Enzyme Ability					
				Antagonism Test ^c^	Chitinase ^d^	Cellulase	Proteases	Glucanase	Siderophores
Surface stem	Tillering	5.5 × 10^6^	60	44	10	6	24	2	2
	Heading	5.4 × 10^6^	40	23	5	4	8	2	4
Interior stem	Tillering	5.5 × 10^5^	86	39	9	3	18	5	4
	Heading	5.4 × 10^5^	30	21	8	3	10	0	0
Endorhiza	Tillering	5.5 × 10^5^	41	34	10	4	12	4	4
	Heading	5.4 × 10^5^	18	12	4	0	7	0	1
Endosphere	Tillering	5.2 × 10^5^	22	18	6	2	8	1	1
	Heading	5.5 × 10^4^	27	18	6	2	8	2	0
Phyllosphere	Tillering	5.4 × 10^5^	80	64	20	9	26	4	5
	Heading	5.6 × 10^4^	25	16	6	0	8	1	1
Rhizophere	Tillering	5.3 × 10^4^	46	28	8	4	10	4	2
	Heading	5.4 × 10^5^	20	4	1	2	10	1	2
Soil	Tillering	5.3 × 10^5^	55	36	16	2	8	4	6
	Heading	5.4 × 10^5^	23	16	5	2	7	1	1

Notes: ^a^ BCAs were screened at two maize growth stages: tillering and heading. ^b^ Bacterial concentration represents the total viable bacteria count (CFU g^−1^) recovered from maize tissues. ^c^ Antagonistic activity was tested using the *Magnaporthe grisea* strain Guy11. ^d^ Isolates producing a visible inhibition halo on WA medium were classified as antagonists; halo width was measured after incubation.

**Table 3:** Summary of enzyme activities by strain source.

Strain Source	Chitinase	Cellulase	Protease	Glucanase	Siderophore
Surface stem	15	10	32	4	6
Interior stem	17	6	28	5	4
Endorhiza	14	4	19	4	5
Endosphere	12	4	16	3	1
Phyllosphere	26	9	34	5	6
Rhizosphere	9	6	20	5	4
Soil	21	4	15	5	7

**Table 4:** Identification and biological control efficacy of bacterial strains isolated from maize samples.

Strains	Identify Results ^a^	Similaraty (%) ^b^	Atangonim	Proteae	Cellulse	Chitinse	Glucanse	Siderophores	Scores		
			Value	Value	Value	Value	Value	Value		FAW Severity (%) ^c^	Biological Control Efficacy (%)
DR-55	*Bacillus subtilis*	100	3	3	1	5	2	1	15	16.67 ± 5.56 ^qr^	88.00%
DR-63	*Enterobacter* sp.	100	2	3	1	4	1	1	12	18.52 ± 3.21 ^qr^	82.00%
HL-7	*Bacillus cereus*	100	2	2	1	2	1	1	9	33.21 ± 4.97 ^op^	75.00%
HL-37	*Bacillus cereus*	99	2	2	0	2	1	0	7	35.19 ± 8.49 ^nop^	55.50%
HR-12	*Enterobacter* sp.	98	2	1	0	2	1	0	6	36.11 ± 2.78 ^mnop^	45.25%
DR-19	*Bacillus cereus*	99	1	1	0	1	0	0	3	45.19 ± 7.14 ^ghijklmn^	32.00%
DL-34	*Bacillus cereus*	99	1	1	0	1	0	0	3	49.81 ± 3.16 ^fghijkl^	32.75%
DR-15	*Bacillus cereus*	99	1	1	0	1	0	0	3	53.7 ± 6.42 ^defghij^	27.50%
DR-8	*Bacillus subtilis*	99	1	1	0	1	0	0	3	54.32 ± 13.4 ^defghi^	26.67%
HL-14	*Bacillus cereus*	99	1	0	0	1	0	0	2	53.02 ± 9.92 ^efghijk^	28.42%
HL-3	*Bacillus subtilis*	99	1	1	0	0	0	0	2	54.07 ± 6.42 ^defghi^	27.00%
HL-22	*Enterobacter* sp.	99	1	0	0	1	0	0	2	46.11 ± 10.2 ^ghijklmn^	24.17%
DR-42	*Bacillus pumilus*	99	1	0	0	1	0	0	2	44.07 ± 5.01 ^hijklmno^	44.50%
DL-26	*Bacillus cereus*	99	1	1	0	0	0	0	2	47.04 ± 0.32 ^ghijklm^	28.50%
DS-6	*Bacillus subtilis*	99	1	0	0	1	0	0	2	50.99 ± 7.86 ^fghijkl^	31.17%
DR-51	*Bacillus subtilis*	100	1	0	0	1	0	0	2	41.67 ± 7.35 ^klmno^	33.75%
DL-2	*Bacillus pumilus*	99	1	0	1	1	0	0	2	43.09 ± 4.69 ^ijklmno^	31.83%
DL-7	*Bacillus cereus*	99	1	0	0	0	1	0	2	41.23 ± 2.04 ^lmnop^	31.33%
DS-5	*Bacillus cereus*	99	1	0	0	1	0	0	2	58.77 ± 7.46 ^cdef^	20.67%
HR-11	*Bacillus cereus*	99	1	0	0	1	0	0	2	50.68 ± 3.26 ^fghijkl^	31.58%
DL-20	*Bacillus subtilis*	99	1	1	0	0	0	0	2	54.94 ± 2.47 ^defgh^	25.83%
DL-21	*Bacillus subtilis*	99	1	0	0	1	0	0	2	52.96 ± 9.71 ^efghijk^	28.50%
DR-3	*Bacillus subtilis*	99	1	0	0	1	0	0	2	64.81 ± 8.49 ^cdef^	12.50%
DL-45	*Bacillus cereus*	99	1	1	0	0	0	1	2	56.67 ± 4.01 ^cdefg^	23.50%
DL-33	*Bacillus cereus*	99	1	0	0	0	1	0	2	54.32 ± 13.4 ^defghi^	26.67%
DL-28	*Bacillus* sp.	99	1	0	0	1	0	0	2	51.23 ± 4.66 ^fghijkl^	30.83%
HR-19	*Bacillus cereus*	99	1	0	0	1	0	0	2	63.7 ± 7.56 ^bcde^	17.50%
HL-37	*Bacillus idriesis*	99	0	0	0	0	0	0	0	65.12 ± 6.17 ^bcd^	12.08%
DS-60	*Bacillus cereus*	99	0	0	0	0	0	0	0	56.51 ± 2.2 ^cdefg^	23.71%
HL-1	*Pantoea* sp.	99	0	0	0	0	0	0	0	53.02 ± 9.92 ^efghijk^	19.25%
HL-20	*Pantoea* sp.	99	1	0	0	1	0	0	2	41.13 ± 2.47 ^mn^	30.46%
HS-8	*Pantoea* sp.	99	0	0	0	0	0	0	0	60.66 ± 1.43 ^b^	13.43%
DL-22	*Acinetobacter* sp.	99	1	1	0	0	0	0	2	60.89 ± 3.49 ^bc^	29.83%
HR-14	*Bacillus cereus*	99	1	0	0	1	0	0	2	31.12 ± 3.35 ^q^	35.51%
DL-26	*Bacillus cereus*	99	0	0	0	0	0	0	0	58.56 ± 3.52 ^cde^	16.58%

Note: ^a^ Identification based on 16S rRNA gene sequencing results. ^b^ Similarity (%) determined by BLAST sequence alignment. ^c^ FAW = Fall Armyworm; severity values represent mean ± standard error. Different superscript letters indicate significant differences (*p* < 0.05) according to Tukey’s test.

**Table 5:** Bacterial isolates showing in vivo biocontrol efficacy against FAW.

Strain	Organism	Score	FAW Severity (% ± SE)	Biocontrol Efficacy (%)	Remarks
DR-55	*Bacillus subtilis*	15	16.67 ± 5.56	88.00	Strong enzyme producer (high chitinase = 5); best control efficacy
DR-63	*Enterobacter* sp.	12	18.52 ± 3.21	82.00	Moderate enzyme profile; excellent efficacy
HL-7	*Bacillus cereus*	9	33.21 ± 4.97	75.00	Effective but lower enzyme activities
HL-37	*Bacillus cereus*	7	35.19 ± 8.49	55.50	Moderate potential

Note: The highest-scoring isolate (DR-55, *Bacillus subtilis*) exhibited the most comprehensive enzymatic arsenal (notably chitinase 5, protease = 3, glucanase = 2), translating to superior FAW suppression (88% efficacy). This suggests synergistic roles of multiple lytic enzymes in degrading insect cuticles and interfering with pest physiology.

**Table 6:** Effects of neem and moringa extracts on *Spodoptera frugiperda* mortality (%) at different stages.

Treatments	Larva (Mean ± SEM)	Pupa (Mean ± SEM)	Adult (Mean ± SEM)
T1	13.36 ± 1.23 ^c^	8.17 ± 0.11 ^b^	2.19 ± 0.01 ^b^
T2	31.32 ± 0.24 ^b^	10.24 ± 0.23 ^b^	3.23 ± 0.01 ^b^
T3	80.13 ± 1.06 ^a^	14.56 ± 1.53 ^a^	6.29 ± 0.53 ^a^
T4	86.23 ± 1.12 ^a^	16.67 ± 1.12 ^a^	6.63 ± 0.49 ^a^
T5	83.11 ± 0.45 ^a^	15.14 ± 0.64 ^a^	6.67 ± 0.51^a^
T6 (Control)	1.13 ± 0.13 ^d^	1.10 ± 0.13 ^c^	0.30 ± 0.06 ^c^

Note: Means ± SEM followed by different superscript letters (a–d) within a column indicate significant differences among treatments according to one-way ANOVA followed by Tukey’s HSD test (*p* < 0.05).

## Data Availability

The datasets generated and/or analyzed during the current study are available from the corresponding author upon reasonable request.
